# Pregnenolone Sulfate Potentiates the Inwardly Rectifying K^+^ Channel Kir2.3

**DOI:** 10.1371/journal.pone.0006311

**Published:** 2009-07-21

**Authors:** Toru Kobayashi, Kazuo Washiyama, Kazutaka Ikeda

**Affiliations:** 1 Department of Molecular Neuropathology, Brain Research Institute, Niigata University, Chuo-ku, Niigata, Niigata, Japan; 2 Division of Psychobiology, Tokyo Institute of Psychiatry, Setagaya, Tokyo, Japan; Yale School of Medicine, United States of America

## Abstract

**Background:**

Neurosteroids have various physiological and neuropsychopharmacological effects. In addition to the genomic effects of steroids, some neurosteroids modulate several neurotransmitter receptors and channels, such as *N*-methyl-D-aspartate receptors, γ-aminobutyric acid type A (GABA_A_) receptors, and σ_1_ receptors, and voltage-gated Ca^2+^ and K^+^ channels. However, the molecular mechanisms underlying the various effects of neurosteroids have not yet been sufficiently clarified. In the nervous system, inwardly rectifying K^+^ (Kir) channels also play important roles in the control of resting membrane potential, cellular excitability and K^+^ homeostasis. Among constitutively active Kir2 channels in a major Kir subfamily, Kir2.3 channels are expressed predominantly in the forebrain, a brain area related to cognition, memory, emotion, and neuropsychiatric disorders.

**Methodology/Principal Findings:**

The present study examined the effects of various neurosteroids on Kir2.3 channels using the *Xenopus* oocyte expression assay. In oocytes injected with Kir2.3 mRNA, only pregnenolone sulfate (PREGS), among nine neurosteroids tested, reversibly potentiated Kir2.3 currents. The potentiation effect was concentration-dependent in the micromolar range, and the current-voltage relationship showed inward rectification. However, the potentiation effect of PREGS was not observed when PREGS was applied intracellularly and was not affected by extracellular pH conditions. Furthermore, although Kir1.1, Kir2.1, Kir2.2, and Kir3 channels were insensitive to PREGS, in oocytes injected with Kir2.1/Kir2.3 or Kir2.2/Kir2.3 mRNA, but not Kir2.1/Kir2.2 mRNA, PREGS potentiated Kir currents. These potentiation properties in the concentration-response relationships were less potent than for Kir2.3 channels, suggesting action of PREGS on Kir2.3-containing Kir2 heteromeric channels.

**Conclusions/Significance:**

The present results suggest that PREGS acts as a positive modulator of Kir2.3 channels. Kir2.3 channel potentiation may provide novel insights into the various effects of PREGS.

## Introduction

Neurosteroids are synthesized by neurons and glial cells in the central and peripheral nervous system from cholesterol or other blood-borne steroidal precursors [1], [2]. In addition to the genomic effects of steroids via intracellular steroid receptors, some steroids modulate the functions of several neurotransmitter receptors and channels, namely γ-aminobutyric acid type A (GABA_A_) receptors, *N*-methyl-D-aspartate (NMDA) receptors, serotonin (5-hydroxytryptamine, 5-HT) subtype 3 (5-HT_3_) receptors, and σ_1_ receptors [3], voltage-gated Ca^2+^ and K^+^ channels [4], [5], and transient receptor potential M3 channels [6], possibly leading to modulation of neuronal excitability. Steroids with these properties are referred to as neuroactive steroids independently of their origin [3]. Neurosteroids have been shown to have a variety of neuropsychopharmacological effects, such as neuroprotective, memory-enhancing, sedative, anxiolytic, sleep-modulating, antidepressant, anticonvulsant and anesthetic effects [2], [3]. However, the molecular mechanisms underlying the various effects of neurosteroids have not yet been sufficiently clarified.

Inwardly rectifying K^+^ (Kir) channels play important roles in the control of resting membrane potential, cellular excitability and K^+^ homeostasis in the nervous system and various peripheral tissues [7]. Among seven Kir subfamilies, members of Kir2 channels in a major Kir subfamily are characterized by strong inward rectification and constitutive activity and are present in various cells, including neurons, glial cells, cardiac and skeletal myocytes, epithelial cells, and macrophages [8]. Four Kir2 channel members have been identified in mammals [9]–[13]. In the nervous system, Kir2.1 channels are expressed widely but weakly in most brain regions. Kir2.2 channels are expressed mainly in the cerebellum. Kir2.3 channels are expressed predominantly in the forebrain [14], [15]. Kir2.4 channels are expressed predominantly in motoneurons of the brainstem [13]. Among Kir2 channels, Kir2.3 channels in the forebrain may be related to cognition, memory, emotion and neuropsychiatric disorders. Therefore, endogenous modulators of Kir2.3 channels may induce various physiological and neuropsychopharmacological effects. In the present study, we investigated the effects of various neurosteroids on Kir2.3 channels using the *Xenopus* oocyte expression assay.

## Results

### PREGS potentiates Kir2.3 channels

**Figure 1:**
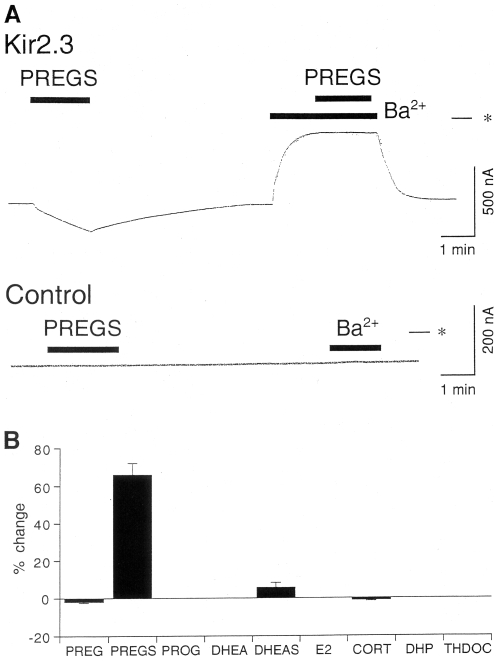
Effects of pregnenolone sulfate (PREGS) on Kir2.3 channels expressed in *Xenopus* oocytes. (A) Upper row, in an oocyte injected with Kir2.3 mRNA, current responses to 30 µM PREGS and to 50 µM PREGS in the presence of 3 mM Ba^2+^ are shown. Lower row, in an uninjected oocyte, no significant current responses to 300 µM PREGS and 3 mM Ba^2+^ are shown. Current responses were measured at a membrane potential of −70 mV in an hK solution containing 96 mM K^+^. Asterisks show the zero current level. Horizontal bars show the duration of application. (B) Effects of various neurosteroids: PREG, PREGS, DHEA, DHEAS, progesterone (PROG), 17β-estradiol (E2), corticosterone (CORT), 3α-OH-DHP and THDOC, on Kir2.3 channels. The magnitudes of the effect of 100 µM neurosteroids on Kir2.3 channels were normalized to the 3 mM Ba^2+^-sensitive current components in oocytes expressing Kir2.3 channels (*n*≥4 for each steroid). Data are expressed as mean±SEM.

In *Xenopus* oocytes injected with Kir2.3 mRNA, inward currents through the expressed Kir 2.3 channels were observed at a holding potential of −70 mV in an hK solution containing 96 mM K^+^ (Fig. 1A). Extracellular application of 30 µM pregnenolone sulfate (PREGS) reversibly potentiated Kir2.3 currents (Fig. 1A). The current responses to an additional 50 µM PREGS during application of 3 mM Ba^2+^, which blocks Kir channels, were not significant (1.5±1.0 nA, less than 1% of the 3 mM Ba^2+^-sensitive current component, *n* = 4; Fig. 1A). The 3 mM Ba^2+^-sensitive current components in oocytes expressing Kir2.3 channels (684.7±79.2 nA, *n* = 25) are considered to correspond to the magnitudes of Kir2.3 currents in oocytes expressing Kir2.3 channels [11]. PREGS produced no significant response in the K^+^-free ND98 solution containing 98 mM Na^+^ instead of the hK solution (5.7±2.0 nA at 50 µM, *n* = 4), suggesting that the PREGS-induced currents show K^+^ selectivity. In uninjected oocytes, 300 µM PREGS and 3 mM Ba^2+^ produced no significant response (less than 5 nA and 3.2±2.5 nA, respectively, *n* = 5; Fig. 1A) compared with oocytes injected with Kir2.3 mRNA, suggesting no significant effect of PREGS and Ba^2+^ on intrinsic oocyte channels. Additionally, application of dimethyl sulfoxide (DMSO), the solvent vehicle, at the highest concentration (0.3%) produced no significant response in oocytes injected with Kir2.3 mRNA (*n* = 4; data not shown). In contrast, 100 µM of the other neurosteroids tested: PREG, dehydroepiandrosterone (DHEA), DHEAS, progesterone, 17β-estradiol, corticosterone, 5α-pregnan-3α-ol-20-one (3α-OH-DHP, also known as allopregnanolone) and 3α, 21-dihydroxy-5α-pregnan-20-one (also known as tetrahydrodeoxycorticosterone, THDOC), produced no significant current responses in oocytes expressing Kir2.3 channels (less than 2% change of the 3 mM Ba^2+^-sensitive current component, with the exception of DHEAS with only 5.6±2.6% potentiation, *n*≥4; Fig. 1B). The results suggest that Kir2.3 channels are potentiated specifically by PREGS among various neurosteroids.

### Characteristics of Kir2.3 channel potentiation by PREGS

**Figure 2:**
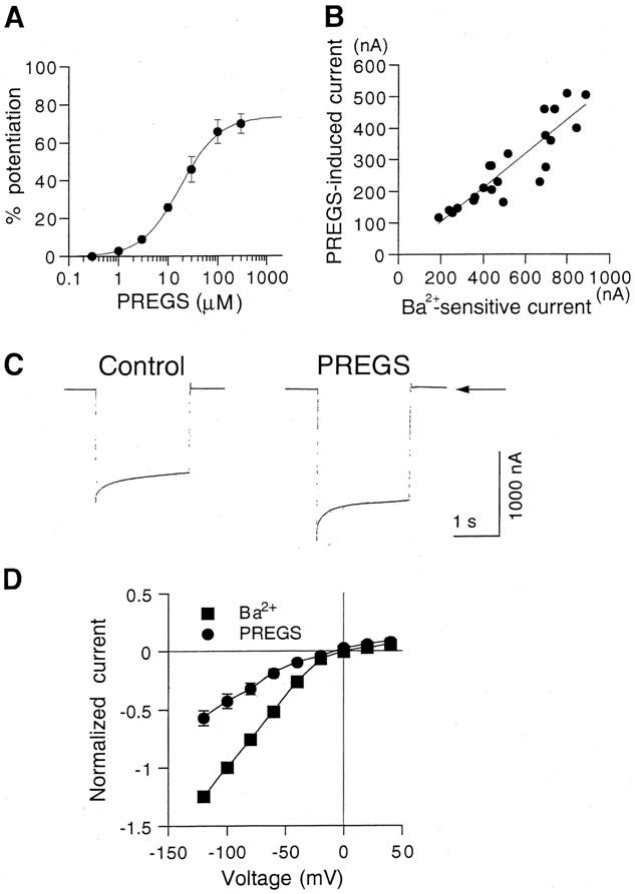
Characteristics of Kir2.3 channel potentiation by PREGS. (A) Concentration-dependent effect of PREGS on Kir2.3 channels. The magnitudes of Kir2.3 currents potentiated by PREGS were normalized to the 3 mM Ba^2+^-sensitive current components in *Xenopus* oocytes expressing Kir2.3 channels (554.0±79.9 nA, *n* = 6). Data are expressed as mean±SEM of the percentage responses. (B) Correlation between amplitudes of current response to 30 µM PREGS and amplitudes of the 3 mM Ba^2+^-sensitive current components in oocytes expressing Kir2.3 channels. The correlation coefficient was 0.894 (*P*<0.05, *n* = 22, regression analysis). Current responses were measured at a membrane potential of −70 mV in an hK solution containing 96 mM K^+^. (C) Representative Kir2.3 currents elicited by a voltage step to −100 mV for 2 s from a holding potential of 0 mV in the presence or absence of 30 µM PREGS in an oocyte injected with Kir2.3 mRNA. Arrow indicates the zero current level. (D) Current-voltage relationships of 3 mM Ba^2+^-sensitive currents and 30 µM PREGS-enhanced currents in oocytes expressing Kir2.3 channels. Current responses were normalized to the 3 mM Ba^2+^-sensitive current component measured at a membrane potential of −100 mV (1510.0±209.9 nA, *n* = 6).

The potentiation of Kir2.3 channels by PREGS was concentration-dependent at micromolar concentrations, with a concentration of PREGS that produces 50% of the maximal effect (EC_50_) of 15.6±0.9 µM and a Hill coefficient (*n*_H_) of 1.43±0.03 (*n* = 6, Fig. 2A). The amplitudes of 30 µM PREGS-potentiated Kir2.3 currents were highly correlated with those of 3 mM Ba^2+^-sensitive current components (Fig. 2B), suggesting that the effects of PREGS may be associated with Kir2.3 channel expression levels.

Furthermore, instantaneous Kir2.3 currents elicited by the voltage step to −100 mV from a holding potential of 0 mV were enhanced in the presence of 30 µM PREGS applied for 3.5 min (Fig. 2C). The percentage potentiation of the steady-state Kir2.3 current at the end of the voltage step by PREGS was not significantly different from that of the instantaneous current (paired *t*-test, *P*>0.05; *n* = 6 at −40, −60, −80, −100 and −120 mV, respectively). These results suggest that the channels were potentiated by PREGS primarily at the holding potential of 0 mV and in a time-independent manner during each voltage pulse. Similarly to 3 mM Ba^2+^-sensitive currents corresponding to Kir2.3 currents, PREGS-induced currents in oocytes expressing Kir2.3 channels increased with negative membrane potentials, and the current-voltage relationships showed inward rectification (Fig. 2D), indicating a characteristic of Kir currents. Furthermore, the PREGS-induced current did not change the reversal potential. These results suggest that PREGS potentiates the function of Kir2.3 channels.

**Figure 3:**
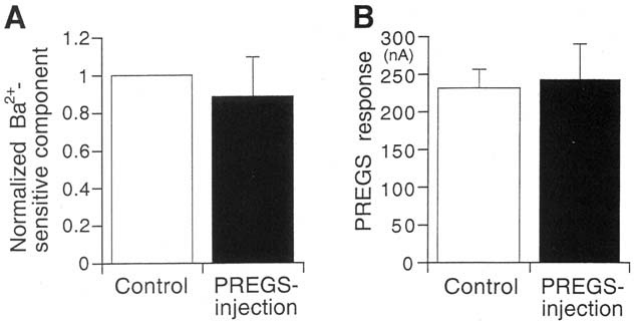
Effect of intracellular PREGS in *Xenopus* oocytes expressing Kir2.3 channels. (A) Comparison of basal Kir2.3 currents before and after PREGS injection in oocytes expressing Kir2.3 channels. The amplitude of Kir2.3 currents was normalized to the amplitude of 3 mM Ba^2+^-sensitive current components before PREGS injection. (B) Comparison of 50 µM PREGS-induced Kir2.3 currents before and after PREGS injection. Data are expressed as mean±SEM.

The effects of intracellular PREGS in oocytes expressing Kir2.3 channels were also examined. The 3 mM Ba^2+^-sensitive current components corresponding to the magnitudes of Kir2.3 currents were not significantly affected by intracellularly applied PREGS (88.4±12.1% of untreated control, *P*>0.05, paired *t*-test, *n* = 4; Fig. 3A), and extracellular application of 50 µM PREGS after the injection similarly potentiated Kir currents (*P*>0.05, paired *t*-test, *n* = 4; Fig. 3B). These results suggest that the potentiation effect of PREGS was not caused by its intracellular action.

**Figure 4:**
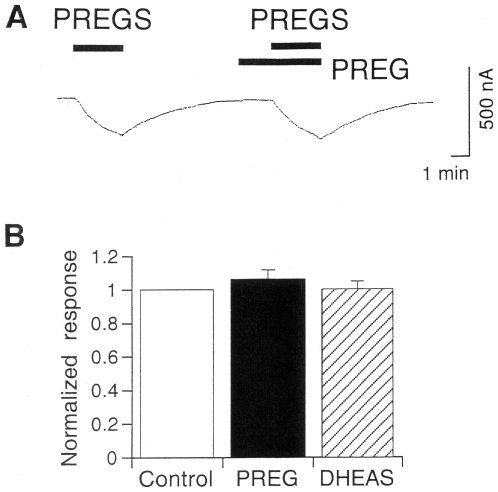
Effects of PREG and DHEAS on PREGS-induced Kir2.3 currents. (A) Representative current responses to 30 µM PREGS and to 30 µM PREGS in the presence of 100 µM PREG in a *Xenopus* oocyte expressing Kir2.3 channels. Current responses were measured at a membrane potential of −70 mV in an hK solution containing 96 mM K^+^. (B) Comparison of PREGS-induced Kir2.3 currents in the presence or absence of PREG or DHEAS. Concentrations of PREGS, PREG, and DHEAS were 30, 100, and 100 µM, respectively. Current responses to PREGS in the presence of PREG or DHEAS were normalized to the amplitude of PREGS-induced currents in the absence of PREG or DHEAS (control). Data are expressed as mean±SEM.

The chemical structure of PREGS shares the structural moiety of PREG and DHEAS [3]. However, 30 µM PREGS-induced Kir2.3 currents were not significantly different from those in the presence of either 100 µM PREG or 100 µM DHEAS (105.9±11.4% and 99.8±8.9% of control, respectively, *P*>0.05, *n* = 4, paired *t*-test; Fig. 4), suggesting that PREG and DHEAS have no significant antagonist effect on the potentiation of Kir2.3 channels by PREGS.

**Figure 5:**
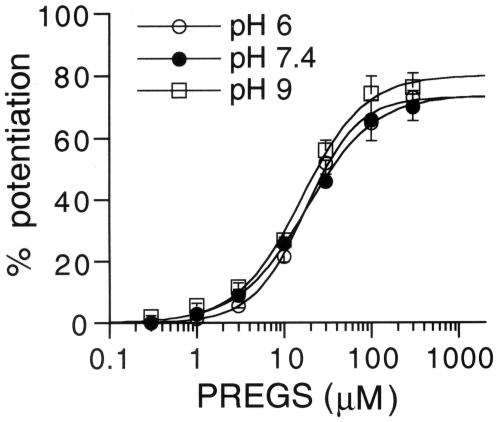
Concentration-response relationships for potentiation of Kir2.3 channels by PREGS at different pH values. The magnitudes of potentiation of Kir2.3 currents by PREGS in oocytes expressing Kir2.3 channels were normalized to the 3 mM Ba^2+^-sensitive current components, which were 426.9.6±41.4 nA (pH 6.0), 554.0±79.9 nA (pH 7.4) and 729.2±36.6 nA (pH 9.0). The EC_50_ and *n*_H_ values were 16.1±1.2 µM and 1.44±0.07 (pH 6.0, *n* = 10), 15.6±0.9 µM and 1.43±0.03 (pH 7.4, *n* = 6), and 17.1±1.5 µM and 0.70±0.03 (pH 9.0, *n* = 8), respectively. Current responses were measured at a membrane potential of −70 mV in an hK solution containing 96 mM K^+^. Data are expressed as mean±SEM of the percentage responses.

Kir2.3 channels are modulated by extracellular pH [16]–[18]. We examined whether changes in pH would alter the effects of PREGS on Kir2.3 channels expressed in *Xenopus* oocytes. In oocytes injected with Kir2.3 mRNA, Kir2.3 currents decreased with a decrease in extracellular pH (51.9±7.9% of the 3 mM Ba^2+^-sensitive current components at pH 7.4 for pH 6.0, *n* = 6; and 167.6±22.1% of those at pH 7.4 for pH 9.0, *n* = 6) as reported in previous studies [16]–[18]. However, the concentration-response relationships for the potentiation effects of PREGS were not significantly affected by pH 6.0, 7.4 and 9.0 (no significant pH × PREGS interaction, *P*>0.05, two-way ANOVA; *P*>0.05 at each concentration, Tukey-Kramer *post hoc* test; Fig. 5). These results suggest that the degree of potentiation of Kir2.3 channels by PREGS may be similar even under pathological pH conditions.

### Selective potentiation of Kir2.3 channels by PREGS

**Figure 6:**
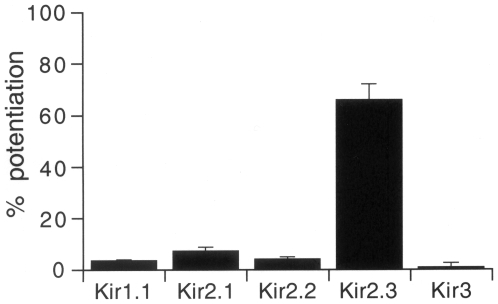
Comparison of the effects of PREGS on Kir1.1, Kir2.1, Kir2.2, Kir2.3, and Kir3 channels expressed in *Xenopus* oocytes. The magnitudes of change in Kir currents by 100 µM PREGS were normalized to the 3 mM Ba^2+^-sensitive current components. For Kir3 channels, oocytes expressing brain-type Kir3.1/Kir3.2 channels were used. Current responses were measured at a membrane potential of −70 mV in an hK solution containing 96 mM K^+^. Data are expressed as mean±SEM.

We also examined the effects of PREGS on other Kir channels (i.e., Kir1.1, an ATP-regulated Kir channel; Kir2.1 and Kir2.2, constitutively active Kir channels; Kir3, a G protein-activated Kir channel [7]). However, in oocytes injected with mRNA for Kir1.1, Kir2.1, Kir2.2, or Kir3.1/Kir3.2 channels, 100 µM PREGS produced no significant current response (3.5±0.4, 7.2±3.7, 4.0±2.5, and 0.8±1.6% change of the 3 mM Ba^2+^-sensitive current components: 820.7±190.8 nA for Kir1.1, 538.0±130.9 nA for Kir2.1, 1518.3±276.4 nA for Kir2.2, and 1043.5±166.0 nA for Kir3.1/Kir3.2, respectively, *n*≥4; Fig. 6). These results suggest that PREGS selectively potentiates Kir2.3 channels.

### Effects of PREGS on Kir2 heteromeric channels

**Figure 7:**
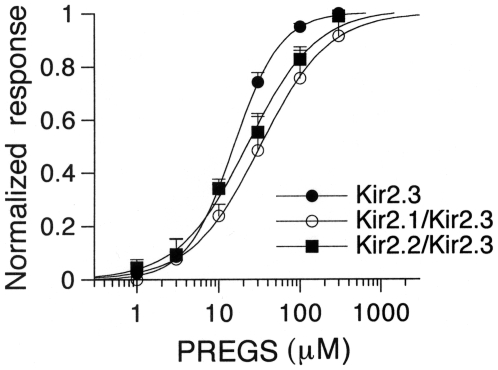
Comparison of the potentiation effects of PREGS on Kir2.3, Kir2.1/Kir2.3, and Kir2.2/Kir2.3 channels expressed in *Xenopus* oocytes. Responses to PREGS at different concentrations were normalized to the maximal response to PREGS. Current responses were measured at a membrane potential of −70 mV. Data are expressed as mean±SEM.

Recent studies have shown that Kir2 channel subunits can form functional heteromeric channels in the *Xenopus* oocyte expression system [19], [20]. We examined the effects of PREGS on Kir2 heteromeric channels. In oocytes injected with mRNA for Kir2.1/Kir2.3 or Kir2.2/Kir2.3, PREGS concentration-dependently potentiated Kir currents (respective EC50 and *n*_H_: 41.0±9.4 µM and 1.40±0.23 for Kir2.1/Kir2.3, *n* = 6; 27.5±5.0 µM and 1.07±0.07 for Kir2.2/Kir2.3, *n* = 9; Fig. 7), whereas PREGS had no significant current response in oocytes injected with Kir2.1/Kir2.2 mRNA (1.3±1.3% inhibition of the 3 mM Ba^2+^-sensitive current components, *n* = 5). The potentiation properties of PREGS in the normalized concentration-response relationships were less potent for Kir2.1/Kir2.3 and Kir2.2/Kir2.3 than for Kir2.3 channels (EC50: *P*<0.05, one-way ANOVA; significant difference between Kir2.1/Kir2.3 and Kir2.3, *P*<0.05, Tukey-Kramer *post hoc* test; and significant channel-type × PREGS interaction, *P*<0.05, two-way ANOVA; *P*<0.05 at 30 µM, Tukey-Kramer *post hoc* test). The results suggest that PREGS may also potentiate Kir2.3-containing Kir2 heteromeric channels.

## Discussion

In the present study, we demonstrated that PREGS potentiated the function of Kir2.3 channels at micromolar concentrations, whereas the eight other neurosteroids (i.e., PREG, DHEA, DHEAS, progesterone, 17β-estradiol, corticosterone, 3α-OH-DHP, and THDOC) had no significant effect on Kir2.3 channels. Kir1.1, Kir2.1, Kir2.2 and Kir3 channels in other members of the Kir channel family were insensitive to PREGS. These results suggest that the endogenous steroid PREGS may selectively act as a positive modulator of Kir2.3 channels. Furthermore, the effect of PREGS on Kir2.3 channels was not caused by intracellularly applied PREGS, and potentiation of Kir2.3 channels by extracellularly applied PREGS was readily reversible with washout. PREGS exists in a negatively charged form, and it could not readily permeate the cell membrane. Therefore, the effect of PREGS was unlikely to be caused by intracellular PREGS or by interactions with intracellular molecules, including phosphatidylinositol 4,5-bisphosphate (PIP_2_) and long-chain fatty acids that activate Kir2.3 channels [21], [22]. Additionally, PREG and DHEAS, whose structures are closely related to PREGS, had no significant effects on Kir2.3 channels or on PREGS-induced Kir2.3 potentiation, suggesting that the effect of PREGS is unlikely to be mediated by a nonspecific membrane-perturbation effect. These observations suggest that PREGS may act directly at Kir2.3 channels, and the site of action of PREGS on Kir2.3 channels may be extracellular or, at least, at a readily accessible site from the outside of the cell membrane. Moreover, the present study suggests that PREGS also potentiates Kir2 heteromeric channels containing Kir2.3 channel subunits. The Kir2.3 channel may be considered as a target site for PREGS.

Administration of PREGS has been shown to have antiamnesic, anxiolytic, antidepressant, neurogenesis, neuroprotective, proconvulsant, and antinociceptive effects [23]–[29] and prevent the development of tolerance and dependence to morphine and benzodiazepines [30], . PREGS at micromolar concentrations has also been shown to modulate the functions of several receptors and channels, namely GABA_A_ receptors, glycine receptors, NMDA receptors, α-amino-3-hydroxy-5-methylisoxazole-4-propionic acid (AMPA) receptors, σ_1_ receptors, voltage-gated Ca^2+^ and K^+^ channels, and transient receptor potential M3 channels [3]–[6], [27]. PREGS can cross the blood-brain barrier [32], and interactions of PREGS with these target sites are proposed to have important implications in the various effects of PREGS. Kir2.3 channels are highly expressed in neurons and some of oligodendroglial cells in the forebrain, such as the olfactory bulb, cerebral cortex, hippocampus and basal ganglia, and spinal cord [14], [15], [33], [34], areas related to cognition, memory, emotion, nociception and drug addiction. Additionally, Kir2.3 channels colocalize with postsynaptic density-95 (PSD-95) in neuronal populations in the forebrain [35] and are localized at the postsynaptic membrane of excitatory synapses in the olfactory bulb [15], suggesting the existence of postsynaptic Kir2.3 channels. In the present study, PREGS potentiated Kir2.3 channels at micromolar concentrations. Because activation of Kir2.3 channels causes membrane hyperpolarization [36], PREGS may decrease excitability of neurons and glial cells in these regions. Furthermore, the distribution of Kir2.1, Kir2.2 and Kir2.3 subunits overlaps in some regions [14], [33], [34], suggesting the partial existence of Kir2 heteromeric channels. The present study suggests that PREGS also potentiates Kir2.3-containing Kir2 heteromeric channels. Therefore, Kir2.3 potentiation by PREGS might be involved in some of the various neuropsychopharmacological effects.

Bulk concentrations of PREGS in brain tissues have been estimated to be in the nanomolar range [37], [38], or even lower [39], [40]. However, the PREG synthase cytochrome P450 side-chain cleavage enzyme and hydroxysteroid sulfotransferase, which converts PREG to PREGS, are expressed in the brain [2], and these enzymes have been shown to colocalize in hippocampal neurons [38], suggesting local synthesis of PREGS. Furthermore, Mameli *et al*. [41] reported that a PREGS-like neurosteroid released from depolarized postsynaptic CA1 neurons increased the frequency of AMPA-mediated miniature excitatory postsynaptic currents via modulation of presynaptic NMDA receptors, with a magnitude equivalent to that caused by exogenously applied PREGS at 17 µM. This effect was blocked by anti-PREGS antibodies. These findings suggest that local concentrations of released PREGS around these neurons might be in the micromolar range. Moreover, brain levels of neurosteroids, including PREGS, have been shown to be elevated under several pathological conditions, such as cerebral ischemia, epilepsy, stress, and drug addiction [3], [42]–[46]. Altogether, Kir2.3 channels in the forebrain, including the hippocampus, might be potentiated by PREGS via paracrine and autocrine mechanisms under such conditions. Additionally, although PREG is structurally related to PREGS, PREG had no significant effect on Kir2.3 channels (Figs. 1B,4). Steroid sulfatase, which converts PREGS to PREG, has been identified in various brain regions [47]–[49]. Conversion between PREGS and PREG by sulfotransferase and steroid sulfatase might regulate the effect of PREGS on Kir2.3 channels in the brain.

Finally, Kir2.3 channels are also expressed in Schwann cells near the nodes of Ranvier in sciatic nerves, cardiomyocytes, and renal cortical collecting duct principal cells [50]–[52]. Typical plasma concentrations of PREGS have been reported to be 0.2 to 0.4 µM, although plasma concentrations of PREGS in some healthy subjects have been reported to be approximately 1 µM [53]. However, plasma PREGS levels can reach micromolar levels during pregnancy and in patients with 21-hydroxylase deficiency [54], [55]. Elevated PREGS levels overlapped with the concentrations that were effective in potentiating Kir2.3 channels in the present study. Additionally, elevated plasma PREGS levels have been observed in patients with anxiety-depressive disorder, alcohol addiction, or hyperthyroidism [56]–[58]. Kir2.3 channels in the peripheral tissues may be potentiated by elevated PREGS concentrations in certain conditions. Potentiation of Kir2.3 channels by PREGS might affect the regulation of K^+^ buffering in peripheral nerves, the control of cardiomyocyte excitability and K^+^ homeostasis in the kidney. Interestingly, PREGS has been identified in sciatic nerves [59], and PREG has been identified in Schwann cells [60]. In sciatic nerves, Kir2.3 channels may also be potentiated by locally released PREGS. Further studies using local administration of PREGS and *in vitro* preparations, such as culture cells and brain slices, may advance our understanding of the physiological and pharmacological effects of PREGS on Kir2.3 channels in the nervous system, heart, and kidney. Kir2.3 channel potentiation may provide novel insights into the various effects of PREGS.

## Materials and Methods

### Compounds

PREG, PREGS, DHEA, DHEAS, progesterone, 17β-estradiol, corticosterone, 3α-OH-DHP, and THDOC were purchased from Sigma-Aldrich (St. Louis, MO, USA). PREGS was dissolved in distilled water or DMSO, and the others were dissolved in DMSO. The stock solution of each compound was stored at −30°C until use. Each compound was added to the perfusion solution in appropriate amounts immediately before the experiments.

### Preparation of specific mRNA

Plasmids containing the entire coding sequences for the mouse Kir2.2 (GenBank accession number: AB035889), Kir2.3, Kir3.1, and Kir3.2 channel subunits were obtained as described previously [61]–[63]. The sequence of amino acids deduced from the nucleotide sequence of C57BL/6NJcl mouse Kir2.2 in the plasmid pSP35T revealed seven amino acids substitutions compared with BALB/c mouse Kir2.2 [12]. However, the substitutions were identical to those of rat Kir2.2, with the exception of a change from Phe to Leu at codon 255 [10]. Additionally, cDNAs for rat Kir1.1 in pSPORT and mouse Kir2.1 in pcDNA1 were generously provided by Drs. Steven C. Hebert and Lily Y. Jan, respectively [9], [64]. The plasmid pSPKir2.2 was linearized by digestion with SacI, and the others were digested with the appropriate enzyme as described previously [9], [62]–[64]. The specific mRNAs were synthesized *in vitro* using the mMESSAGE mMACHINE™ *In Vitro* Transcription Kit (Ambion, Austin, TX, USA).

### Oocyte electrophysiology

*Xenopus laevis* oocytes (Stages V and VI) were isolated from adult female frogs (Copacetic, Soma, Aomori, Japan) that were anesthetized by immersion in water containing 0.15% tricaine (Sigma-Aldrich) as described previously [65]. All procedures for the care and treatment of animals were approved by Niigata University Institutional Animal Care and Use Committee in accordance with the National Institutes of Health guidelines. Oocytes were injected with mRNA for Kir1.1 (1 ng), Kir2.1 (0.5 ng), Kir2.2 (0.5 ng), Kir2.3 (1 ng), Kir2.1/Kir2.2, Kir2.1/Kir2.3, Kir2.2/Kir2.3 (each 0.5 ng), or Kir3.1/Kir3.2 combinations (each 0.3 ng) for brain-type Kir3 channels. Oocytes were incubated at 19°C in Barth's solution after treatment with 0.8 mg/ml collagenase and manually defolliculated. Whole-cell currents of the oocytes were recorded from 2 to 7 days after the injection with a conventional two-electrode voltage clamp [62], [66]. The membrane potential was held at −70 mV, unless otherwise specified. Microelectrodes were filled with 3 M KCl. The oocytes were placed in a 0.05 ml narrow chamber and superfused continuously with a high-K^+^ (hK) solution (composition in mM: KCl 96, NaCl 2, MgCl_2_ 1, CaCl_2_ 1.5 and HEPES 5, pH 7.4 with KOH) or a K^+^-free high-Na^+^ (ND98) solution (composition in mM: NaCl 98, MgCl_2_ 1, CaCl_2_ 1.5 and HEPES 5, pH 7.4 with NaOH). In the hK solution, the K^+^ equilibrium potential was close to 0 mV, and the inward K^+^ current flow through the Kir channels was observed at negative holding potentials [9], [11], [12], [62], [64]. For examining the effects of intracellular PREGS, 13.8 nl of 10 mM PREGS dissolved in distilled water containing 5% DMSO was injected into an oocyte using a Nanoliter injector (World Precision Instruments, Sarasota, FL, USA) as described previously [67]. The oocyte currents were then continuously recorded for approximately 30–40 min. Because the volume of the oocyte was approximately 1 µl, the intracellular concentration of PREGS was presumed to be approximately 136 µM. Furthermore, injection of the same volume of the solvent vehicle had no significant effect on Kir2.3 currents (*n* = 4). For analysis of concentration-response relationships, data were fitted to a standard logistic equation using KaleidaGraph (Synergy Software, Reading, PA, USA). EC_50_ and *n*_H_ values were obtained from the concentration-response relationships.

### Data analyses

Data are expressed as mean±SEM, and *n* is the number of oocytes tested. Statistical analysis of differences between groups was performed using paired *t*-test, one-way analysis of variance (ANOVA), or two-way ANOVA followed by Tukey-Kramer *post hoc* test. Values of *P*<0.05 were considered statistically significant.
